# Association between oral variables and heart rate variability

**DOI:** 10.1186/1755-7682-6-49

**Published:** 2013-12-27

**Authors:** Milana Drumond Ramos Santana, Ana Cecilia Amorim de Souza, Luiz Carlos de Abreu, Vitor E Valenti

**Affiliations:** 1 Laboratório de Escrita Científica, Faculdade de Medicina do ABC, Av. Príncipe de Gales, 821, Santo André, SP 09060-650, Brazil; 2 Departamento de Morfologia e Fisiologia, Faculdade de Medicina do ABC, Av. Príncipe de Gales, 821, Santo André, SP CEP: 09060-650, Brazil

**Keywords:** Dentistry, Autonomic nervous system, Cardiovascular system

## Abstract

The heart rate variability is a useful method to assess cardiac autonomic modulation in patients undergoing dental procedures, because knowledge of physiological conditions provides greater security to the professional as well as the possibility of a better plan treatment to patient benefit. The aim of our study was to describe the association between cardiac autonomic control and dental variables. We consulted the databases Medline, SciELO, Lilacs and Cochrane, using the terms “autonomic”, “dentistry”, “heart rate variability”, “cardiovascular physiology.” The selected studies indicated a strong relationship between dental variables and HRV. There was an association between malocclusion, TMD, dental procedures cirugia and low HRV. Thus, they become more studies that relate to HRV in dental science, especially in clinical practice.

## Background

The control of the cardiovascular system is accomplished in part by the autonomic nervous system (ANS), which is composed by the sympathetic and parasympathetic pathways that command the cardiovascular system by releasing neurotransmitters that increase or decrease heart rate (HR), respectively. The periodic oscillations in HR and RR intervals of consecutive heart beats, modulated by the activity of ANS on the heart is known as heart rate variability (HRV) [1-5].

The study of HRV has helped to detect and characterize some situations in which diseases affect the autonomic control. A high HRV is a sign of good adaptation, featuring a healthy individual, with efficient autonomic mechanisms, whereas low variability is often an indicator of abnormal and insufficient adaptation of the ANS, implying the presence of physiological malfunction in the individual [4]. The sympathetic branch increases HR, promoting reduced RR intervals and decreasing heart rate variability. On the other hand, decreases in the parasympathetic decreases HR and increases RR intervals and HRV [2].

Within the last decade, HRV has been widely used in clinical research, as it provides quantitative information about the modulation of cardiac activity of vagal and sympathetic nerve. The autonomic nervous system plays a central role in maintaining the stability of hemodynamics and HRV has been recognized as a powerful stratifying risk for adverse cardiac events [6].

Thus, the study of HRV allowed, in a non-invasive, safe and reproducible way to assess the neural control of the heart [3-5].

According Montebugnoli et al. [6], dental treatment, particularly dental surgery, increases sympathetic activity of the heart, in some cases, may trigger adverse cardiac effects. Therefore, HRV can be modified in situations of stress, such as a dental appointment, and studies suggest that it may be a sensitive marker of quantitative autonomic activity during stress. Moreover, most dental treatments are performed under local anesthesia, and increases in blood pressure were reported during tooth extraction even in patients with normal blood pressure [7].

The knowledge of physiological responses involved in oral treatment is important for providing an appropriate treatment plan for the patient, and, thus, increased security to the dentist. Therefore, the aim of our study was to describe the association between cardiac autonomic control and oral variables.

## Methods

We consulted the following databases: Medical Literature Analysis and Retrieval System Online (MEDLINE), Scientific Electronic Library Online (SciELO), Latin American and Caribbean Health Sciences (LILACS) and The Cochrane Library (Cochrane), using terms: “autonomic”, “dentistry”, “heart rate variability”, “cardiovascular physiology” in English and Portuguese. We considered all available years until January 2013.

The studies were selected by a reviewer and supervised by a senior reviewer. Based on titles and abstracts, we excluded manuscripts not clearly related to the subject under review. All titles and abstracts selected underwent a final evaluation, which considered the inclusion criteria. Inclusion criteria were considered evaluation studies of heart rate in dental science.

## Results

The electronic search yielded a total of 131 references. Among these references, the first elimination resulted in the exclusion of 123 titles and abstracts, which were not clearly related to the purpose of the review. The resulting 08 items were submitted to a final evaluation that took into account the inclusion criteria, with 04 selected studies. Table 1 shows the objectives and main findings of the studies included in this research.

**Table 1:** Studies regarding the relationship between cardiac autonomic control and dental science

**Authors and year**	**Objectives**	**Key findings**
Ekuni et al. [10]	To investigate the relationship between malocclusion and HRV in young healthy adults.	Orthodontic treatment can contribute to not only improvement of oral function and aesthetics, but also for the improvement of stress levels and HRV.
Maixner et al. [11]	Check the association between autonomic variables and TMD.	TMD was associated with higher heart rate, reduced HRV and baroreflex sensitivity reduction. These results provide evidence of an association between TMD and autonomic factors.
Montebugnoli et al. [6]	To evaluate the sensitivity of HRV in dental surgery compared with other clinical parameters most frequently used in clinical practice dentistry.	Among the clinical parameters (systolic blood pressure, diastolic blood pressure and HRV) HRV was the only one that was significantly different in all four test periods. Thus, HRV is a highly sensitive parameter for quantifying autonomic impulses to the heart during dental surgery.
Matsumura et al. [12]	To determine changes in blood pressure, pulse and HRV during dental surgery.	The patients of middle age and older presented higher blood pressure increase during dental surgery than younger patients and the regulation of the autonomic nervous system during dental surgery differs between younger and older patients.

TMD: Temporomandibular disorder; HRV: heart rate variability.

## Discussion

Considering that the ANS plays an important role in regulating physiological processes of the human body both under normal and pathological conditions, we aimed to describe the relationship between HRV and the variables related to dentistry. Among the techniques used in its evaluation, HRV has emerged as a simple and non-invasive measure of autonomic impulses, representing one of the most promising quantitative markers of autonomic balance [4].

Patient assessment in an integrated way has become a consensus across dental field. Knowledge of physiological conditions provides greater safety to the practitioner, and also the possibility of a better treatment plan for the patient’s benefit. Thus, we should appreciate the systemic status of the individual, which may produce various disorders that may influence dental treatment, affecting the general welfare [8].

According to Ferraz et al. [8] dental treatment in the body may trigger a series of phenomena that determine the elevation of blood pressure and heart rate, promoting psychosomatic changes that are able to induce hypertensive crisis, compromising the function of vital organs and cause accidents of unexpected proportions.

In this context, HRV may be a useful parameter to detect even small cardiovascular changes than other noninvasive tools for patients undergoing dental procedures. Thus, it can be a useful method to detect heart failurerelated to the local high sympathetic activity, and to prevent cardiovascular emergencies [6].

Dental procedures, particularly the extraction of impacted third molars, can become extremely uncomfortable and painful, causing anxiety, fear and important changes in autonomic patient. The study by Braga et al. [9] concluded that hemodynamic and respiratory changes can occur during extraction of impacted third molars. Thus, the electronic monitoring enables the dentist to diagnose these early changes, and as a consequence decrease the morbidity and mortality in this type of procedure.

For Montebugnoli et al. [6] blood pressure should not be considered as a reliable index to quantify cardiovascular activity during dental surgery. The researchers identified the HRV as one of the most promising marker of cardiac activity. In that study, there was no difference in systolic and diastolic blood pressure between all four study periods (early in the dental surgery, immediately after anesthesia, during tooth extraction and five minutes after tooth extraction). However, the HR values were significantly different in three of the four periods, and the values of HRV were significantly different between all four test periods. Thus, HRV is a highly sensitive parameter for quantifying autonomic impulses to the heart during dental surgery.

The HRV indices have been investigated to understand various conditions, including coronary artery disease, heart failure, hypertension, diabetes, chronic obstructive pulmonary disease, renal failure, epilepsy, depression, among others [4,5,10]. In dentistry, HRV has been associated with malocclusion [10], temporomandibular joint dysfunction [11], in dental surgery [12] and effects of sedatives [13,14]. Ekuni et al. [10] evaluated the effects of malocclusion on the HRV indices. The authors found that malocclusion causes chronic stress that can affect the HRV indices. They suggested that orthodontic treatment may contribute not only to improve aesthetic and functional oral, but also for the improvement of stress levels and HRV. However, the authors suggested that further studies are needed to compare the quality of life and HRV in people who suffer from malocclusion before and after orthodontic treatment.

Maixner et al. [11] investigated the association between autonomic variables and temporomandibular disorder (TMD), testing the hypothesis that dysregulation of the autonomic nervous system contributes to the onset and persistence of TMD. The authors found that patients with TMD at rest showed reduced HRV compared with the control group. According to the authors, the study design does not show whether the change in autonomic activity can be a cause or consequence of the TMD.

Matsumura et al. [12] assessed the changes in blood pressure, pulse and HRV during dental surgery. The study included 40 patients, 19 to 74 years old. Holter electrocardiogram was used to determine the power spectrum of HRV before and during dental surgery. After administration of local anesthetic (2% lidocaine) containing epinephrine 1:80,000, both blood pressure and heart rate increased. The increase in blood pressure was higher in middle-aged and elderly patients (> or = 40 years old). In younger patients (<40 years old), heart failure decreased and LF/HF ratio, which corresponds to the sympathetic-parasympathetic balance, increased during local anesthesia. In contrast, patients in middle-aged and older aged, the LF/HF ratio decreased during local anesthesia. The authors concluded that patients of middle age and older age have a greater increase in blood pressure during dental surgery than younger patients, and that the regulation of the autonomic nervous system during dental surgery differs between younger and older patients.

Most dental treatments are performed under local anesthesia, and increases in blood pressure were reported during the same tooth extraction in patients with normal blood pressure [7]. When dental treatment comes to cardiac patients with ventricular arrhythmia, the scarcity of objective information in the literature makes it difficult to choose the most appropriate anesthetic and the decision on the maximum dose to be used. However, in the study of Caceres et al. [15] it was found that the effects of local anesthetics without vasoconstrictor or with non-adrenergic on the cardiovascular system are not significant.

Miura et al. [7] evaluated changes in blood pressure, pulse rate and HRV during dental surgery in hypertensive patients. The authors concluded that suppression of cardiac sympathetic nervous system during dental surgery may attenuate the response in patients with hypertension. Comparative studies have shown some results that indicate a strong relationship between dental and HRV variables.

## Conclusions

In summary, HRV is a very sensitive parameter to quantify cardiac autonomic modulations and considering the importance of knowing the physiological condition of the patient by the dentist, it is necessary further studies that relate HRV in dental science, especially in clinical practice, i.e. in meeting the various dental specialties. The present review showed an association between malocclusion, TMD, surgical dental procedures and low HRV, thus, strengthening the relationship between variables related to dentistry and HRV.

## Competing interests

The authors declare that they have no competing interests.

## Authors’ contributions

All authors participated in the acquisition of data and revision of the manuscript. All authors determined the design, interpreted the data and drafted the manuscript. All authors read and gave final approval for the version submitted for publication.
